# Gastrointestinal parasite infection intensity and hematological parameters in South African communal indigenous goats in relation to anemia

**DOI:** 10.14202/vetworld.2020.2226-2233

**Published:** 2020-10-27

**Authors:** Takalani Judas Mpofu, Khathutshelo Agree Nephawe, Bohani Mtileni

**Affiliations:** Department of Animal Sciences, Tshwane University of Technology, Pretoria, South Africa

**Keywords:** hemoglobin concentration, mean corpuscular hemoglobin, packed cell volume, strongyles

## Abstract

**Aim:**

The study was conducted to determine the intensity of gastrointestinal parasite (GIP) infections and hematological parameters in South African communal indigenous goats in relation to anemia.

**Materials and Methods:**

A total of 288 goats were randomly sampled in areas representing four agro-ecological zones. Fecal and blood samples were collected from the rectum and jugular vein, respectively, of each animal. The number of eggs per gram (EPG) and oocysts per gram (OPG) of feces and the hematological parameters were determined using the modified McMaster technique and a BC-2800Vet® automatic hematology analyzer, respectively. Data were analyzed using the repeated measures techniques of Minitab 17, modeling the covariance structure of the observed data.

**Results:**

Based on EPG and OPG, goats in humid zone were significantly infected (p<0.05) with strongyles, *Eimeria*, *Moniezia*, and *Trichuris* spp. Hematological parameters of goats in arid and humid zone were lower (p<0.05) than those in semi-arid and dry sub-humid zone. GIP infection intensities were higher (p<0.05) in young animals than in adult and suckling goats. GIP infection intensity was similar between goat sexes, while hematological parameters were higher (p<0.05) in females. Higher (p<0.05) infection intensities for strongyles (302.90 EPG) and *Eimeria* (216.09 EPG) were observed in winter compared to summer (strongyles: 302.90, *Eimeria*: 216.09 EPG). Higher (p<0.05) values for the hematological parameters were observed during summer compared to that in winter.

**Conclusion:**

GIP infection intensity in the winter could be associated with hypochromic and normocytic anemia which likely to affects suckling goats while in the summer could be associated with normochromic and normocytic anemia which likely to affect young goats.

## Introduction

Domestic goats (*Capra* *hircus*) play an important role in meeting the nutritional, economic, and social needs of many rural households [1,2]. The global demand for sources of animal protein is growing [3,4] and it is obvious that the livestock industry, especially goat production, will need to be expanded. However, goats are highly susceptible to gastrointestinal parasite (GIP) infections [5-7], which imposes serious productivity constraints in marginal systems, resulting in clinical diseases, loss of productivity [8,9], and death [10]. GIP control methods previously proposed include chemical treatment, husbandry management, or biological approaches [11]. Of the approaches, chemotherapy (anthelmintic drugs) has been the most widely used method; however, this noticeably has often been associated with cases of drug-resistant parasites [12-14] and drug residues in the end-product food and the environment [15]. The presence and prevalence of GIPs in goats have been well documented in several studies, including goats of South Africa [7,16,17] and other African countries such as Nigeria [18], Egypt [19], Ethiopia [20], Cameroon [21], Ghana [22], and Zimbabwe [23].

Some GIPs suck blood (especially strongyles) and where there is a heavy parasitic burden, they may cause anemia [24] as well as other hematological and biochemical disturbances [25,26]. Anemia is not a disease entity, but rather is functionally defined as decreased oxygen-carrying capability of blood [27]. Clinically, anemia is characterized by an absolute decrease in the packed cell volume (PCV), hemoglobin concentration (Hgb) [28], and total erythrocyte count per unit volume of blood with values below the normal generally observed in hydrated animals [29]. PCV, Hgb, and mean corpuscular hemoglobin (MCH) are major indices used in the diagnosis of anemia [30]. Typically, PCV values in goats ranges between 22% and 38% [31,32], whereas the Hgb and MCH range 8-18 pg/dL and 5.2-8 pg, respectively [32]. Animals with lower hematological values are considered anemic [30,33]. Animals with good blood composition are likely to show good production performance [34]. Hematological studies are of ecological and physiological interest to understand the relationship of blood characteristics to the environment [35] and to certain diseases [34,36]. Of noteworthy, hematological parameters are dependent on the environment and condition of the animal, such as infection status, breed, age, and other factors. In South Africa, there is a wide range of environments [37,38] with arid and semi-arid agro-ecological zones (AEZs) occupying the largest percentage of land surface area. However, there is currently a paucity of published information available on the intensity of GIPs in South African communal indigenous goats in different AEZs.

The current research was, therefore, conducted to determine the intensity of the GIP infections and the hematological parameters in South African communal indigenous goats in relation to anemia.

## Materials and Methods

### Ethical approval

The study was approved by the Animal Research Ethics Committee of the Faculty of Science, Tshwane University of Technology (FCRE 2017/10/01 [02] [SCI]). Ethical concerns were addressed by adhering to local animal welfare regulations and practices and by conforming to the ethical guidelines of Tshwane University of Technology for animal use in research.

### Study site and animal management

A longitudinal study was conducted in four different AEZs of South Africa: Arid, semi-arid, humid, and dry sub-humid zones. The selected AEZs varied in rainfall distribution and in the percentage of land coverage (Table-1) [38-40]. A total of 288 communal indigenous goats (101 males and 187 females) were randomly sampled during the summer (November-December 2017) and winter (June-July 2018) in areas representing four AEZs: Arid=80; semi-arid=76; humid=62, and dry sub-humid=70. The sample size was determined using Equation 1 [41].


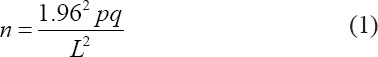


**Table 1:** Agro-ecological zones and their features in South Africa.

Agro-ecological zone	Annual rainfall (mm)	Length of growing period (d)	Aridity index* (P/Ep)	Percentage of land surface	Vegetation type	% Rangeland	% Cultivated
Desert	<200			22.8			
Arid	201-400	<90	<0.39	24.6	Annual grassland	87	7
Semi-arid	401-600	90-179	0.40-0.79	24.6	Thorny savannahs	54	35
Dry sub-humid	601-800	180-269	0.80-0.11	18.5	Broad-leaved savannah woodlands	34	47
Humid	801-1000	270-365	>0.12	6.7	Rain forest and savannahs		
Super humid	>1000			2.8			

* The ratio of precipitation to potential evapo-transpiration; Adapted from Schulze [38]; Mpofu *et al*. [39]; UN, Environment Management Group [40]

where *n*=sample size, *p*=expected prevalence, *q*=1−*p*, and *L*=limits of error on the prevalence. Because the prevalence in the local goat population was unknown, a hypothesized prevalence of 75% was used with a 5% limit of error of the prevalence. Based on these assumptions, the required sample size was calculated to be 288 goats for each set of the seasons. The animals were maintained under extensive grazing systems in which during the day they were released to graze on communal lands and corralled at night. The flocks were classified by age (adult: ≥3 years, young goat: 1-2 years, and suckling kids: <1 year), as described by Kheirandish *et al*. [42]. Plastic ear tags (Allflex® - Somerset West, Western Cape, South Africa) bearing individual identification numbers were placed on the right ear of each animal during the initial sample collection to allow for repeated sampling of the same animals over the study period.

### Sample collection and analysis

Approximately 10 g of fecal material and 10 ml of blood were collected from the rectum and the jugular vein, respectively, of the animals. The samples were collected twice during each season from each of the animals. Fecal samples were placed into airtight containers and labeled while the blood samples were collected by venipuncture into EDTA Vacuette^®^ (Greiner Bio-One, Austria) tubes and labeled. All samples were maintained at 2-4°C in cooler boxes and transported to the laboratory for coprological and hematological examination within 24 h. The fecal samples were subjected to quantitative examination for GIP eggs and oocysts. The eggs per gram (EPG) and oocysts per gram (OPG) counts were determined using a modified McMaster technique as described by Hansen and Perry [43]. For positive fecal samples, slides were prepared for examination under the microscope (10× magnification). The egg floatation fluid used was NaCl (NaCl, 500 g; water, 1000 mL) into a routine fecal floatation test kit (Ovatector® - Kyron Laboratories [Pty] Ltd, South Africa). To prevent bubbles when counting the eggs and oocysts, the fecal samples were ground in five drops of Bloat Guard. Each fecal count was multiplied by 100 to estimate the number of eggs in the entire animal system [44]. The GIPs eggs were identified under a compound microscope (10×) based on morphological appearance and the size of the helminth eggs, protozoa cysts, and trophozoites [45,46]. Distinguishable nematodes (*Trichuris* spp. and *Strongyloides papillosus*), trematodes, and cestode eggs were directly identified. A sample was considered positive when a minimum of one GIP egg was detected under the microscope. Fecal cultures were prepared by incubating 2-3 g of feces at 26-28°C for 7 days with 80% humidity after which time the infecting larvae were collected using a modified Baermann technique. L_3_-stage nematodes were identified according to the protocol proposed by Van Wyk *et al*. [47]. *Eimeria* species were identified following oocyst sporulation within the feces after incubation for 7 days at 26-28°C in a thin layer of 2.5% potassium dichromate. Identification of the *Eimeria* species was based on morphological characteristics of the oocysts (size, shape, color, and presence or absence of a micropyle and its cap). The hematological parameters evaluated included PCV (%), Hgb (g/dL), and MCH (pg), which were analyzed using a BC-2800Vet® Auto Hematology Analyzer (Shenzhen Mindray Bio-Medical Electronics Co., Ltd, Hamburg, Germany).

### Statistical analysis

The EPG and OPG values for all the detected GIPs were transformed through a base 10 logarithm [log_10_ Fecal Egg Count (FEC) + 25] to approximate a normal distribution. The transformed data were used for statistical analysis. The infection intensity and hematological parameters data were analyzed using the repeated measured techniques of the Minitab [48] in PROC MIXED considering the covariance structure of the observed data. The FEC-transformed data and the results were then back-transformed by taking the anti-logarithms and the results were presented as geometric means. The means were separated using Fisher’s protected least significant difference test (p<0.05). The following statistical model was used (Equation 2).

*Y_ijklm_*=μ+*T_i_*+*ε_ij_*+*W_k_*+*S_l_*+*D_m_*+*ε_ijklm_* (2)

Where *Y*_ijkl_=measurement of response (individual GIP species FEC, hemoglobin, PCV, and MCH) on the *j*^th^ animal, μ=overall mean, *T_i_*=fixed effect of AEZ (arid, semi-arid, dry sub-humid and humid), *W_k_*=fixed effect of the *k*^th^ season on measurements (*k*=summer and winter), *S_l_*=fixed effect of the *l*^th^ sex on measurements (*l*=male and female), *D_m_*=fixed effect of the *m*^th^ age on measurements (*m*=suckling, young and adult goat), *ε_ij_*=random effect associated with the *j*^th^ animal on the *i*^th^ AEZ, *ε_ijklm_*=random error associated with the *j*^th^ goat of a *l*^th^ sex and *m*^th^ age at the *k*^th^ season in the *i*^th^ AEZ.

## Results

The L_3_ nematodes identified in the fecal cultures for all the animals in arid, semi-arid, and humid zones were *Haemonchus* spp., *S. papillosus*, and *Oesophagostomum* spp. In the dry sub-humid zone, *Haemonchus* and *S. papillosus* were the L_3_ nematodes that were identified. Regarding *Eimeria* species, *E. arloingi*, *E. christenseni*, *E. alijevi*, *E. jolchijevi*, *E. caprina*, *E. caprovina*, and *E. hirci* were identified in fecal cultures from all AEZs. Five species of GIP eggs and oocysts observed were *Moniezia*, *S. papillosus*, *Trichuris*, *Eimeria* spp., and strongyles with mean EPG counts of 179.53, 182.70, 195.00, 201.69, and 274.45, respectively (Table-2). The mean MCH, Hgb, and PCV for South African communal indigenous goats in the present study were 4.97 pg, 8.71 g/dl, and 27.03%, respectively. The AEZ significantly influenced the intensity of all the GIP infections and hematological blood parameters (p<0.05). The infection intensities of strongyles (323.50EPG), *Eimeria* (263.60OPG), *Moniezia* spp. (194.04OPG), and *Trichuris* spp. (236.00EPG) were significantly higher in the humid-zone goat populations compared to that in other zones (*p*<0.05). Based on EPG and OPG values, lower infection intensities of the GIPs understudy were observed in arid (strongyles, 218.70; *S. papillosus*, 151.01; *Moniezia* spp., 158.11; and *Trichuris*, 180.49EPG) and semi-arid (*Eimeria*, 167.29OPG) zones. For hematological parameters, Hgb, PCV, and MCHC of goats in arid zones (Hgb, 8.19 g/dl; PCV, 25.84%; and MCH, 4.28 pg) and humid zones (Hgb, 8.12; PCV, 25.63; MCH, 4.53 pg) were significantly lower (p<0.05) compared to those in semi-arid zones (Hgb, 9.17 g/dl; PCV, 28.12%; and MCH, 5.47 pg) and dry sub-humid zones (Hgb, 9.36 g/dl; PCV, 28.54%; and MCH, 5.60 pg).

**Table 2:** Mean number of gastrointestinal parasite eggs per gram (±SE) of fecal material and hematological parameters for goats in different agro-ecological zones of South Africa.

Parameters	Agro-ecological zones				Mean

	Arid	Semi-arid	Dry sub-humid	Humid	
Gastrointestinal parasites					
*Eimeria* spp.	169.96^c^±10.7	167.29^c^±10.9	205.89^b^±11.3	263.60^a^±12.2	201.69
*Trichuris* spp.	180.49^b,c^±10.5	165.73^c^±10.7	197.76^b^±11.1	261.00^a^±11.9	195.00
*Strongyloides papillosus*	151.01^c^±10.9	195.60^a,b^±11.10	211.70^a^±11.40	172.50^b,c^±12.30	182.70
*Moniezia* spp*.*	158.11^b^±7.83	208.83^a^±7.98	157.12^b^±8.23	194.04^a^±8.86	179.53
Strongyles	218.71^c^±14.0	275.00^b^±14.3	280.60^b^±14.8	323.50^a^±15.9	274.45
Mean FEC	181.65	178.72	227.66	233.83	
Hematological parameters					
Hemoglobin (g/dl)	8.19^b^±0.21	9.17^a^±0.21	9.36^a^±0.22	8.12^b^±0.23	8.71
PCV (%)	25.84^b^±0.47	28.00±12^a^±0.48	28.54^a^±0.50	25.63^b^±0.54	27.03
MCH (pg)	4.28^b^±0.20	5.47^a^±0.20	5.60^a^±0.21	4.53^b^±0.23	4.97

^a,b,c^Row means with different superscripts differs significantly (p<0.05); FEC=Fecal egg count, PCV=Packed cell volume, MCH=Mean corpuscular hemoglobin

The GIP infection intensity was significantly higher (p<0.05) in young animals (strongyles, 455.2; *S. papillosus*, 317.9; *Eimeria*, 330.3; *Moniezia* spp., 325.10; and *Trichuris*, 315.62EPG) compared to that in suckling kids (strongyles, 86.10; *S. papillosus*, 39.50; *Eimeria*, 80.30; *Moniezia* spp., 36.70; and *Trichuris*, 71.04EPG) and adult goats (strongyles, 282.04EPG; *Eimeria*, 194.51; and *Moniezia* spp., 176.77OPG) (Table-3). The infection intensity was similar for *S. papillosus* (317.90 vs. 190.83) in young and adult goats (p>0.05); however, the adult and suckling goats were also similarly infected with *Eimeria* (194.51 vs. 80.30OPG). For hematological parameters, Hgb, PCV, and MCH were significantly different (p<0.05) for goat ages, with the highest mean values being recorded in adult goats (Hgb, 9.66 g/dl; PCV, 29.27%; and MCH, 5.82 pg), followed by young goats (Hgb, 8.71 g/dl; PCV, 27.02%; and MCH, 5.08 pg), and lastly by suckling kids (Hgb, 7.76 g/dl; PCV, 24.87%; and MCH, 4.02 pg).

**Table 3:** Mean gastrointestinal parasite eggs per gram (±SE) of fecal material and hematological parameters between goats of different ages.

Parameters	Goat ages		

	Suckling	Young	Adult
Gastrointestinal parasites			
*Eimeria* spp.	80.30^b^±13.6	330.30^a^±12.2	194.51^b^±7.07
Trichuris spp.	71.04^c^±13.3	315.62^a^±11.9	196.84^b^±6.94
*Strongyloides papillosus*	39.50^c^±13.7	317.9^a^±12.30	190.83^a^±7.16
Moniezia spp.	36.70^c^±9.90	325.10^a^±8.87	176.77^b^±5.15
Strongyles	86.10^c^±17.8	455.2^a^±15.9	282. ±04^b^±9.24
Mean FEC	62.73	348.82	208.20
Hematological parameters			
Hemoglobin (g/dl)	7.76^c^±0.26	8.71^b^±0.23	9.66^a^±0.14
PCV (%)	24.87^c^±0.60	27.02^b^±0.54	29.26^a^±0.31
MCH (pg)	4.02^c^±0.25	5.08^b^±0.23	5.82^a^±0.13

^a,b,c^Row means with different superscripts differs significantly (p<0.05); FEC=Fecal egg count; PCV=Packed cell volume, MCH=Mean corpuscular hemoglobin

The infection intensity of the *Eimeria* and strongyles was significantly influenced by season (p<0.05), but the influence of season on infection intensity of *S. papillosus*, *Moniezia*, and *Trichuris* spp. was insignificant (p>0.05) (Table-4). Significantly higher infection intensities were observed for strongyles (302.90EPG) and *Eimeria* (216.09OPG) during winter season compared to that in summer season (p<0.05), whereas, the *S. papillosus*, *Moniezia*, and *Trichuris* spp. infection intensities were similar between seasons (p>0.05). There was a significant effect of season on hematological blood parameters (p<0.05) with higher values being observed during the summer season (Hgb, 10.08 g/dl; PCV, 28.52%; and MCH, 5.47 pg) compared to that in the winter season (Hgb, 7.34 g/dl; PCV, 25.52%; and MCH, 4.47 pg).

**Table 4:** Mean gastrointestinal parasite eggs per gram (±SE) of fecal material and goat hematological parameters in different seasons and goat sexes.

Parameters	Season		Sex	
	
	Winter	Summer	Female	Male
Gastrointestinal parasites				
*Eimeria* spp.	216.09^a^±8.39	187.27^b^±8.39	201.34^a^±8.14	202.03^a^±9.19
*Trichuris* spp.	203.35^a^±8.23	185.64^a^±8.23	186.45^a^±7.99	202.54^a^±9.02
*Strongyloides papillosus*	192.29^a^±8.49	173.19^a^±8.49	174.35^a^±8.24	191.13^a^±9.30
*Moniezia* spp.	185.95^a^±6.11	173.10^a^±6.11	258.58^a^±5.93	100.47^b^±6.70
Strongyles	302.90^a^±11.0	246.00^b^±11.0	260.50^a^±10.6	266.40^a^±12.0
Mean FEC	220.11	193.04	202.24	206.57
Hematological parameters				
Hemoglobin (g/dl)	7.34^b^±0.16	10.08^a^±0.16	9.33^a^±0.16	8.09^b^±0.18
PCV (%)	25.58^b^±0.37	28.52^a^±0.37	28.50^a^±0.36	25.60^b^±0.41
MCH (pg)	4.47^b^±0.16	5.47^a^±0.16	5.62^a^±0.15	4.32^b^±0.17

^a,b,c^Row means with different superscripts differs significantly (p<0.05); FEC=Fecal egg count, PCV=Packed cell volume, MCH=Mean corpuscular hemoglobin

There was no significant influence from the sex of the goats on any of the GIP infections (p>0.05), except for *Moniezia* spp. for which higher infection intensity values were observed in female goats (258.58) compared to those in male goats (100.47OPG). The hematological parameters were significantly higher (p<0.05) in female goats (Hgb, 9.33 g/dl; PCV, 28.50%; and MCH, 5.62 pg) compared to that in male goats (Hgb, 8.09 g/dl; PCV, 25.60%; and MCH, 4.32 pg).

## Discussion

In this paper, and to be consistent with epidemiological terminologies, the intensity of the infection is defined as the mean number of EPG or OPG of feces calculated over all samples tested positive for the parasite, intensity is zero when a host has no GIP [49,50]; this contrasts with the definition suggested by Bush *et al*. [51]. Anemia can be classified according to cell size (normocytic, microcytic, or macrocytic, indicating normal, increased, or decreased PCV, respectively) and to Hgb concentration (normochromic, hypochromic, or hyperchromic, indicating normal, decreased, or increased Hgb concentration, respectively) [52,53].

The findings that infection intensity for all GIPs and hematological parameters significantly differed with AEZ (p<0.05) may be attributed to differences in AEZ’s climatic factors, such as temperature, rainfall pattern, aridity index, and humidity, which are known to influence infection intensity and the development of nematode eggs [23,54,55]. Higher infection intensity for all of the GIPs, except for *S. papillosus* in humid zones, may be associated to the fact that the humid areas experience warm temperatures and high rainfall (801-1000 mm annually), which are capable of sustaining plant life for 270-365 plant growing days [38,56]. This, in turn, provides a favorable environment for pre-parasitic nematodes development, survival, and transmission [43,54].

The higher infection intensity of *Moniezia* spp. observed in semi-arid goat populations could be attributed to shorter rainy seasons and higher temperatures [38,57], which may be conducive for *Moniezia* spp. development or to increased availability of intermediate parasite hosts in the vicinity [58]. The lower infection intensity observed for strongyles, *S. papillosus*, *Moniezia*, *Eimeria*, and *Trichuris* spp. in arid zone may have been attributed to warmer temperatures and low erratic rainfall levels [38,57], which are unfavorable for the GIP development, survival, and transmission [43,56]. However, in contrast to our current findings, a lack of variation in GIP infection intensity across different locations has also been reported [54,58].

Lower hematological parameters for goats in humid zones could be associated with the high infection intensities of GIPs in those areas. These results are in agreement with several previous reports that low PCV [59,60], Hgb [61], and MCH [30] values are commonly associated with high FEC, attributed to the adult parasites sucking a substantial amount of blood from the abomasum of infected animal. Despite lower GIP infection intensity in arid goat population, noteworthy, the lower hematological parameters observed in this goat population may have been resulted from the nutritional stress associated with these areas as they are characterized by warm temperatures, low erratic rainfall distribution [56], and fluctuations in forage quality and quantity. Nutritional stress is known to lower the hematological parameters of animals [62-64]. The higher hematological parameters for goat populations in the semi-arid and dry sub-humid zones can be a result of lower GIP infection intensity. For the goats in the AEZs evaluated in the current study, infection intensity and hematological parameters (PCV and Hgb) suggested that GIP infections resulted to normocytic and normochromic anemia, indicating normal values for PCV and Hgb concentration.

Infection intensity revealed significant differences between age groups (*p*<0.05), with young animals having higher EPG or OPG compared to that of adult and suckling animals. These findings are in agreement with the previous reports where young animals tend to be more susceptible to infections [23,65,66]. The increased susceptibility of young animals may be due to immunological immaturity and immunological unresponsiveness [67]. In contrast, adult animals may acquire immunity to parasites through frequent challenges and thereby expel ingested parasites before an infection is established [68]. Contrary to our current findings that suckling and adult goats were equally infected with *Eimeria* (p>0.05), Verma *et al*. [57] reported that goats between 1 and 6 months of age (suckling kids) are heavily infected with *Eimeria* compared to those of goats older than 12 months of age (adults). The observed difference in suckling, young, and adult South African communal indigenous goats suggests that the oxygen-carrying capacity of the blood was higher in adult goats, which can be supported by the fact that immunological maturity is acquired with increasing age after repeated exposure [57]. Suckling animals have lower PCV, Hgb, and MCH in comparison with adult animals [69]. It may be postulated that GIP infection intensity resulted in hypochromic and normocytic anemia in suckling goats, while normochromic and normocytic anemia resulted in young and adult goats.

The effect of the sex of animals was not significant (p>0.05) except for the infection intensity of *Moniezia* spp., where mean OPG value was significantly higher in females compared to that in males (p<0.05). A lack of significant effect of the sex of animals on infection intensity for several GIPs had also been previously reported [57] and may have been attributed to the fact that both male and female goats are exposed to similar environmental conditions that are conducive for GIP infection. However, the current findings are in contrast with some other reports that claim the sex of the animals have a significant association with the infection intensity of GIP [19]. Ayaz *et al*. [66] reported that the intensity of infection is higher in male animals compared to that in female animals, while Sharma *et al*. [65] reported higher infection intensity in females compared to that in males. Specifically, high *Moniezia* spp. infection intensities in females might be due to stress and low immune status during pregnancy, parturient paresis, or lactation periods [55]. The findings that hematological parameters were significantly higher in females may be attributed to the fact that females are more resistant to GIP [69]. The GIP infection intensity across the sex of the goat resulted in normochromic and normocytic anemia.

The findings that animals during the winter season were more heavily infected with *Eimeria* and strongyles compared to that in the summer season was noteworthy since one could anticipate that *Eimeria* arthropod intermediate hosts would favor a moist microclimate in the pasture. Higher GIP infection intensity in wet months (summer season) compared to winter months has been reported by various researchers [65,70] and is attributed to the differences in the humidity and temperature during summer season, which is favorable for the development, hatching, survival, and translocation of pre-parasitic stages. This might result in higher infection intensities during the summer season. However, reduced grazing hours also reduces the chances of contact between hosts and parasites, leading to lower infection intensities in winter season. Inclement environmental conditions in winter season force strongyles to go under hypobiosis, resulting in a reduction in the number of eggs [57]. The higher hematological parameters observed during summer may have been due to the lower infection intensities of *Eimeria* and strongyles, while the lower hematological parameters observed during winter may also have been associated with higher infection intensities of *Eimeria* and strongyles in the same season. Higher infection intensities of strongyles lead to the lower PCV and Hgb concentrations in animals [71]. The GIP infection intensities in winter resulted in hypochromic and normocytic anemia, which may have resulted from higher infection intensities by strongyles and *Eimeria*, while in the summer season the GIP infection intensities resulted in normochromic and normocytic anemia.

## Conclusion

GIP infection intensity varies with AEZs and the age of goat, and, may affect the health status and productivity of goats. The results from the current study suggest that the GIP infection intensity in the winter season resulted in hypochromic and normocytic anemia, whereas in the summer season and across the sex of goat resulted in normocytic and normochromic anemia. These hematological parameters can be used as indicators of anemia resulted from the GIP infections in goat. Further studies are recommended to investigate the genetic resistance against GIP in South African communal indigenous goats using the intensity of infection and hematological parameters as phenotypic indicators.

## Authors’ Contributions

This study is the component of the work toward the Ph.D. thesis of the first author TJM, under the guidance of KAN and BM. TJM designed the study, participated in all experiments, coordinated the data analysis and wrote the manuscript. KAN and BM designed the study, coordinated all the work and revised the manuscript. All authors read and approved the final manuscript.
